# Adenotonsillectomy - immediate post operative respiratory distress

**DOI:** 10.1016/S1808-8694(15)30503-6

**Published:** 2015-10-19

**Authors:** Denise Manica, Mariana Magnus Smith

**Affiliations:** 1 ENT Resident Physician - Porto Alegre University Hospital; 2 MSc in Pediatrics - UFRGS. ENT Physician - Porto Alegre University Hospital

**Keywords:** adenoidectomy, apnea, pulmonary edema, tonsillectomy

## INTRODUCTION

Post-obstructive pulmonary edema, a kind of non-cardiogenic pulmonary edema^1^, is a rare disorder. Such condition was first described in humans in 1973^2^ and its incidence is difficult to precise since most of the studies are case reports. Two distinct mechanisms have been described in the literature. Type I is the one that follows an acute airway obstruction, such as a laryngospasm, which can happen after any surgical procedure, being more common after otorhinolaryngological surgeries^3^. Type II happens after releasing a chronic upper airway obstruction, being considered less common than type I^4^. We hereby, report on a patient submitted to adenotonsillectomy which evolved with immediate post-operative respiratory dysfunction.

## CASE PRESENTATION

**Figure 1:**
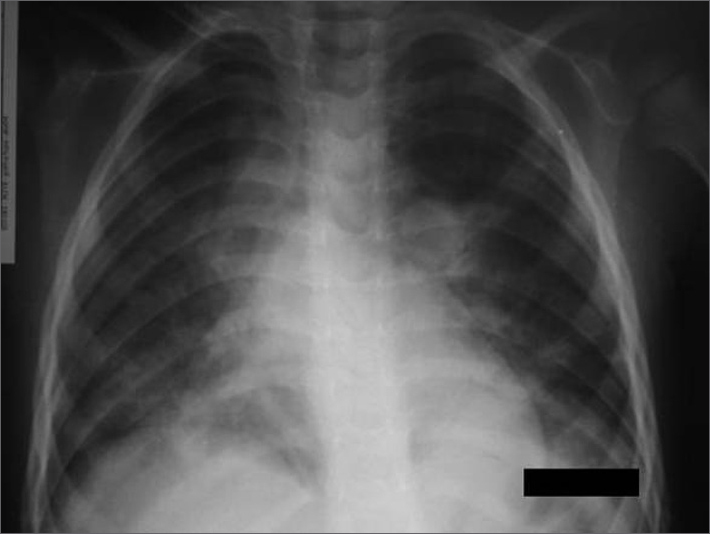
Chest X-Ray during respiratory dysfunction, showing the bilateral interstitial infiltrate.

Male, two years and 8 months old, seen at the pediatric emergency ward due to respiratory dysfunction. His mother reported a past of snoring and sleep apnea for 1 year, which worsened in the 2 weeks prior to the visit. During physical exam, the physician noticed a greater inspiratory ventilation effort, with subcostal retraction. Oxygen saturation was kept between 98 and 100% while awake, which dropped down to 82% during sleep. He had palatine tonsil hypertrophy grade 45, and normal pulmonary auscultation. The patient was submitted to adenotonsillectomy, which happened without complications. In the immediate post-op the child was restless, with low oxygen saturation, without responding to oxygen therapy through a nasal catheter. There was no stridor, nor any other sign of upper respiratory obstruction. The patient was placed in a Venturi mask and nebulized with adrenaline, with an improvement in his breathing pattern. A chest X-Ray showed a diffuse interstitial pulmonary infiltrate, without heart enlargement (Fig. 1). The patient required ventilatory assistance for about 24 hours. Another chest X-Ray taken two days later was normal.

## DISCUSSION

This patient's clinical history strongly suggested a diagnosis of type II postobstructive pulmonary edema, since there was a chronic obstruction of the airway which was quickly solved by surgery without signs of upper airway obstruction. Type II pulmonary edema is explained by the final positive expiratory pressure generated by the chronic obstructed airway. When it is relieved, the sudden reduction in intrathoracic pressure increases venous return, thus increasing hydrostatic pressure and creating edema^6^. In the type I, the breathing effort needed to surpass the acute obstruction creates a negative pressure that is enough to increase blood volume, raising hydrostatic pressure and giving rise to the edema^6^.

Both types of edema have clinical presentations similar to that of respiratory dysfunction, tachypnea, tachycardia, drop in oxygen saturation, snoring and crackling sounds at pulmonary auscultation. Nonetheless, these presentations may present themselves as an isolate and asymptomatic radiological finding^4^.

The treatment used for our patient was monitoring at the pediatric ICU and administration of supplementary oxygen, which is in agreement with recommendations present in the literature^4^,^6^. The infusion of liquids must be careful and diuretics should be used judiciously as well. Steroids can be useful considering the alveolar damage in the edema pathogenesis. Full edema resolution usually happens within 24 hours^3^, which was what happened in this clinical case.

## FINAL COMMENTS

It is fundamental that physicians who treat pediatric patients with chronic airway obstruction have knowledge on the possibility of the patient developing post-obstructive pulmonary edema after surgery. Although this is a self-limited complication most of the times, only requiring monitoring and oxygen supplementation, a rapid onset can cause a potentially fatal complication if not promptly recognized and acted upon.
